# Supplementary material for: Hexavalent Chromium Inhibits Nitrate-Dependent Anaerobic Methane Oxidation While Enriching Denitrifiers: Insights into Microbial Interactions for Simultaneous Methane, Nitrate, and Chromate Removal
Source: ACS ES T Water. 2025 Oct 2;5(11):6790–800. doi: 10.1021/acsestwater.5c00752 (PMC12624737; doi:10.1021/acsestwater.5c00752)
Supplement: Supplementary file 1 [file ew5c00752_si_001.pdf]

Supplement Information

Hexavalent Chromium Inhibits Nitrate-Dependent Anaerobic Methane Oxidation While Enriching Denitrifiers: Insights into Microbial Interactions for Simultaneous Methane, Nitrate and Chromate Removal

Yinxiao Ma, Garrett Smith, Suzanne S.C.M. Haaijer-Vroomen, Sanne Olde Olthof, Cornelia U. Welte, Martyna Glodowska

Calculation of the total amount of $^{13}CO_2$ and $^{15}N_2O$

$$\sum {^{13}CO_2} = {^{13}CO_{2(g)}} [1 + kRT\ V_{liquid}/V_{gas} (1+ K_Z/[H^+])] \qquad \text{Equation S1}$$

Where:

1) $\sum {^{13}CO_2}$ is the total amount of $^{13}CO_2$ in the bottle

2) $^{13}CO_{2(g)}$ is the amount of $^{13}CO_2$ in the gas phase (headspace) in mmol.

3) k is the solubility coefficient of CO_2, which is 3.3×10^{-4} mol/m^3 Pa.

4) R is the universal gas constant, from the ideal gas law, which is 8.314 J mol^{-1} K^{-1}.

5) T is the Kelvin temperature of incubation condition. 4ºC = 277.15 K, RT is ~22 ºC = 295.15 K

6) V_{liquid} and V_{gas} are the volumes of the liquid and gas phases (in mL).

7) K_Z is the dissociation constant of the first step of carbonic acid dissociation. That is K_{a1}, which you get from pK_1, which you have to calculate (see below).

8) [H^+] is the molar concentration of H^+

Gibbs Free energy for Cr(VI) reduction couple to NO_2^- Oxidation

$$Cr_2O_7^{2-}+6NO_2^{-}+14H^+ \rightarrow 2Cr^{3+}+6NO_3^{-}+7H_2O.$$

$$\Delta G=\sum \Delta G_f^\circ(\text{products})-\sum \Delta G_f^\circ(\text{reactants}).$$

$\Delta G_f^\circ(Cr_2O_7^{2-})= -1301$kJ/mol $\Delta G_f^\circ(NO_2^-)= -229$kJ/mol $\Delta G_f^\circ(H^+)=0$kJ/mol $\Delta G_f^\circ(Cr^{3+})=-378$kJ/mol $\Delta G_f^\circ(NO_3^-)=-372$kJ/mol $\Delta G_f^\circ(H_2O)=-237.1$kJ/mol

Data from Chemistry WebBook (https://webbook.nist.gov/) and Thermochemical Tables (https://atct.anl.gov/)

Net $\Delta G = \Delta G_f^\circ(\text{products}) - \Delta G_f^\circ(\text{reactants})$

$=-4647.7-(-2675)$

$=-4647.7+2675$

$=-1972.7$kJ/mol

328 kJ/mol per one mole of NO_3^-

ΔG is negative ($\Delta G=-1972.7$ kJ/mol < 0), and the reaction is **spontaneous** under standard conditions.

Calculation of redox potential for Cr(III)/Cr(VI) couple

$$Cr_2O_7^{2-} + 6NO_2^- + 14H^+ \rightarrow 2Cr^{3+} + 6NO_3^- + 7H_2O$$

the electric potential of $Cr_2O_7^{2-}/Cr^{3+}$ is +1.33V (E°=+1.33V) (Ng et al., 2022)

When pH = **7**, the proton concentration is: $[H^+]=10^{-7}M$

The Nernst equation for this reaction is:

$$E = E^0 - \frac{0.0591}{N} * \log\left(\frac{[Cr^{3+}]^2}{[Cr_2O_7^{2+}]*[H^+]^{14}}\right)$$

N= 6 (number of transferred electrons)

Assuming standard concentrations for ions (1M)

$$\log\left(\frac{[Cr^{3+}]^2}{[Cr_2O_7^{2+}]*[H^+]^{14}}\right) = \log\left(\frac{1}{[H^+]^{14}}\right) = \log\left(\frac{1}{(10^{-7})^{14}}\right) = 98$$

$$E = 1.33v - \frac{0.0591}{6} * 98 = 0.365v$$

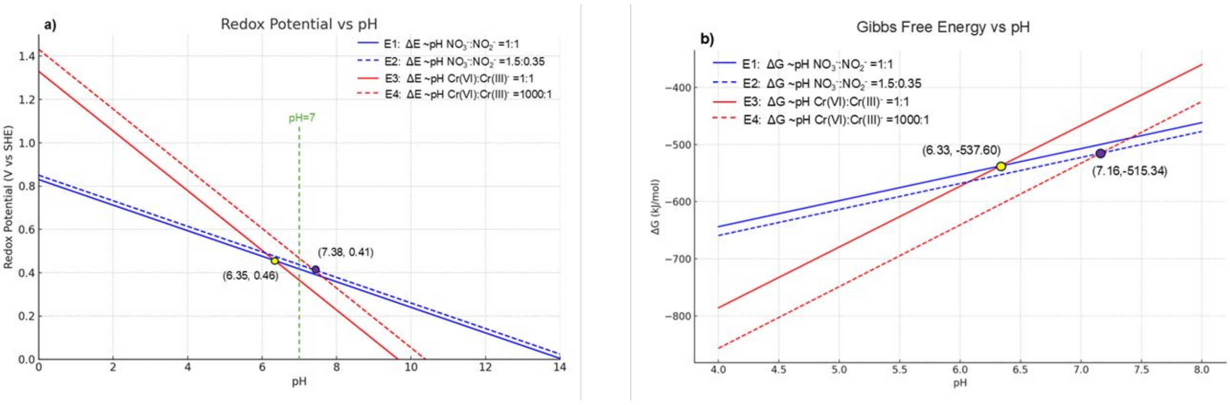

Figure S1. The change of a) redox potential and b) Gibbs free energy with pH in standard biological and experimental conditions. The red solid and dashed lines represent the Cr(VI)/Cr(III) couple at concentration ratios of 1:1 and 1000:1, respectively. The blue solid and dashed lines represent the NO_3^-/NO_2^- couple at concentration ratios of 1:1 and 1.5:0.35, respectively

Calculation details for Fig. S1

$Cr_2O_7^{2-} + 14H^+ + 6e^- \rightarrow 2Cr^{3+} + 7H_2O$

$E_{Cr(VI)/Cr(III)} = E^0 - \frac{0.0591}{N} * \log\left(\frac{[Cr^{3+}]^2}{[Cr_2O_7^{2+}]*[H^+]^{14}}\right)$ $E_{Cr(VI)/Cr(III)}^0 = +1.33V$ N=6 (Ng et al., 2022)

$NO_3^- + 2H^+ + 2e^- \rightarrow NO_2^- + H_2O$

$E_{NO_3^-/NO_2^-} = E^0 - \frac{0.0591}{N} * \log\left(\frac{[NO_2^-]^1}{[NO_3^-]*[H^+]^2}\right)$ $E_{NO3-/NO2-}^0 = +0.83V$ N=2 (Bhattarai et al., 2019)

In standard biological conditions, [ox] = [red], therefore,

$$E_{NO_3^-/NO_2^-} = E^0_{NO_3^-/NO_2^-} - 0.059 * pH$$

E$_1$: $\boldsymbol{E_{NO_3^-/NO_2^-} = 0.83v - 0.059 * pH}$

when pH=7, $E_{NO3-/NO2-} = E^{0'}_{NO3-/NO2-} = 0.417V$

which is close to the previous calculation by Liebensteiner et al., 2014

$$E_{Cr(VI)/Cr(III)} = E^0_{Cr(VI)/Cr(III)} - 0.1378 * pH$$

E$_3$: $\boldsymbol{E_{Cr(VI)/Cr(III)} = 1.33v - 0.1378 * pH}$

when pH=7, $E_{Cr(VI)/Cr(III)} = E^{0'}_{Cr(VI)/Cr(III)} = 0.365v$

In experimental condition, here we took the concentration of 0.3mM treatment (26h) as an example, $[NO_3^-]:[NO_2^-]=1.5:0.3$, Cr(VI):Cr(III)=1000:1

E$_2$: $\boldsymbol{E_{NO_3^-/NO_2^-} = 0.85v - 0.059 * pH}$

E$_4$: $\boldsymbol{E_{Cr(VI)/Cr(III)} = 1.43v - 0.1378 * pH}$

Similarly, according to $\Delta G = - nFE$, we can then derive the relationship between Gibbs free energy and pH under various conditions. According to Eq.1 and Eq.2, n=8, $\Delta E_{CH4}^{0'} = -0.24v$ and F=96485C/mol, therefore:

ΔG$_1$: $\Delta G_{NO3-/NO2-} = -N*F*(E_{NO3-/NO2-} - E_{CH4})$

$$= -8 * 96.5 * (0.83v - 0.059 * pH - (-0.24v))$$

$$= -826.04 + 45.55 * pH$$

Similarly:

ΔG$_3$: $\boldsymbol{\Delta G_{Cr(VI)/Cr(III)} = -\ 1212 + 106.5 * pH}$

ΔG$_2$: $\boldsymbol{\Delta G_{NO_3^-/NO_2^-} = -\ 841.5 + 45.55 * pH}$

ΔG$_4$: $\boldsymbol{\Delta G_{Cr(VI)/Cr(III)} = -1289.24 + 106.5 * pH}$
